# Genetic landscape of hypertrophic cardiomyopathy in Hong Kong Chinese population

**DOI:** 10.3389/fgene.2025.1583838

**Published:** 2025-05-16

**Authors:** Derek P. H. Lee, Ye Cao, Lilei Zhang

**Affiliations:** ^1^ Department of Medicine, Queen Elizabeth Hospital, Kowloon, Hong Kong SAR, China; ^2^ Department of Obstetrics and Gynaecology, The Chinese University of Hong Kong, Hong Kong, Hong Kong SAR, China; ^3^ Department of Molecular and Human Genetics, Baylor College of Medicine, Houston, TX, United States

**Keywords:** hypertrophic cardiomyopathy, cardiomyopathy, Genetic landscape, population genetics, Hong Kong, Chinese

## Abstract

**Introduction:**

Asian populations are underrepresented in the hypertrophic cardiomyopathy (HCM) genomic databases, which are currently largely dominated by Caucasian population. We aim to characterize the genetic landscape of HCM in patients from Hong Kong Chinese population.

**Methods:**

From March 2023 to March 2024, fifty-three unrelated patients with an unequivocal clinical diagnosis of HCM were enrolled at a single tertiary center in Hong Kong and underwent genetic testing using a standardized 19-gene panel.

**Results:**

In this cohort study, we identified 13 patients (24.5%) with a predominant pathogenic or likely pathogenic (P/LP) variant and 12 patients (22.6%) with a predominant variant of unknown significance (VUS). Most of the P/LP variants identified were in *MYBPC3* (46.2%, n = 6) or *MYH7* (38.5%, n = 5). Novel genetic variants were identified in 5 patients. Multiple genetic variants identified in the same patient were common (13.2%, n = 7). All disease-causing variants are rare with allele frequencies <0.00005 in all populations and <0.0002 in East Asian subpopulation. Specifically in this unrelated cohort, we identified several recurrent variants including *MYH7*:c.1987C>T (p.Arg663Cys) pathogenic missense variant (n = 2), *MYBPC3*:c.1038_1042dup (p.Met348Thrfs*4) pathogenic truncating variant (n = 3) and *MYBPC3*:c.1000G>A (p.Glu334Lys) missense VUS (n = 3). Patients with P/LP variants were associated with an increased risk of developing left ventricular dysfunction (p = 0.012).

**Conclusion:**

Our study provided insight into the genetic landscape of HCM in Hong Kong Chinese population. We identified several recurrent variants and novel variants in our HCM cohort. Patients with P/LP variants were associated with an increased risk of developing left ventricular dysfunction. Future studies on the potential founder effects of these recurrent variants, cumulative effects of multiple variants, and longitudinal follow up of HCM patients would be useful.

## 1 Introduction

The Asia-Pacific region, particularly in low- and middle-income countries, has historically faced challenges in the development of clinical genetic services (Thong et al., 2018). These barriers include variable socioeconomic status, diverse cultural differences, lack of awareness in genetic healthcare, issues with gender selection and genetic ethics, as well as religious controversies. However, the increasing availability of advanced sequencing technologies and growing expertise in the field has made clinical genetics more accessible in the region, fostering a rapid expansion in research opportunities and clinical applications (Chung et al., 2019). Despite these advancements, the lack of ethnic diversity in genomic databases is a widely recognized issue. This lack of representation can result in variant misclassifications and genetic misdiagnoses leading to inferior clinical outcomes (Manrai et al., 2016). To address this gap, there is a pressing need for the development of additional genomic databases that incorporate data from diverse ethnicities within the Asia-Pacific region.

Hypertrophic cardiomyopathy (HCM) is estimated to affect 1 in 500 individuals (Maron et al., 1995). However, prevalence rates in the Asia-Pacific region appear to differ from historical global estimates. A recent study conducted in Korea estimated the prevalence in 2016 to be 0.031%, based on data from the Korean National Health Insurance Services database. Though this represents an increase from 0.016% in 2010, it remains significantly lower than the previously reported global prevalence of 0.2% (Moon et al., 2020). HCM is characterized by left ventricular (LV) hypertrophy, defined by wall thickness ≥15 mm in one or more myocardial segments without abnormal loading conditions or other cardiac and systemic causes of hypertrophy, such as congenital heart disease and aortic stenosis (Task Force et al., 2014). Clinical presentation ranges from cardiac arrhythmias and thromboembolic events to congestive heart failure and sudden cardiac death. Despite decades of research into the understanding of genetic landscape in HCM, most of the knowledge came from western genomic databases with predominantly Caucasian population (Marstrand et al., 2020; Neubauer et al., 2019). We aim to provide an insight into the genetic landscape of HCM in Hong Kong Chinese population. We also summarized relevant publications regarding the genetic landscape of HCM within the Chinese population (Table 1).

**TABLE 1:** List of publications on genetic landscape of HCM in Chinese population.

Study	Sequencing method	No. of genes tested	Major findings
Sun et al. (2023)	Whole exome sequencing	25 genes analyzed. (500 additional candidate genes evaluated)	11.3% rare truncating variants identified. Mostly identified in *MYBPC3*. Other variant types, e.g., missense, in the established HCM-associated genes were not reported
Pua et al. (2020)	Targeted re-sequencing	15 genes	18% diagnostic yield and an excess of VUS in 24%
Xu et al. (2015)	Whole exome sequencing	43 genes	Additional 9.5% diagnostic yield in cohort previously determined to be negative for mutations in 8 sarcomere genes
Liu et al. (2013)	Sanger sequencing	3 genes (*MYH7, MYBPC3, TNNT2)*	27% diagnostic yield
Song et al. (2005)	Sanger sequencing	3 genes (*MYH7, MYBPC3, TNNT2)*	34% diagnostic yield

## 2 Methods

### 2.1 Study subjects

A cohort study was conducted in a tertiary center in Hong Kong from March 2023 to March 2024. We recruited 53 unrelated adults (aged 18 or above) Chinese patients with a clinical diagnosis of HCM, which is defined as LV hypertrophy with wall thickness ≥15 mm in one or more myocardial segments without abnormal loading conditions or other cardiac and systemic causes of hypertrophy, such as congenital heart disease and aortic stenosis. Patients with or without family history of HCM were both included. All study participants had undergone cardiac MRI with parametric mapping (specifically T1) and/or echocardiography with imaging evidence of HCM. All patients with unexplained LV hypertrophy were screened for alpha-galactosidase-A level in male and lysoGb3 in female to exclude Anderson-Fabry disease. Additionally, the GLA gene was included in the NGS panel to exclude variants in this gene. Patients with clinical suspicion of infiltrative heart disease were screened for serum monoclonal proteins, free light chains and technetium pyrophosphate scintigraphy. Patients found to have Anderson-Fabry disease, cardiac amyloidosis, syndromic HCM, hypertensive heart disease or severe aortic stenosis were excluded.

Genetic data and clinical information were collected. This study was approved by the Institutional Review Board and was conducted in full compliance with the International Council for Harmonization E6 guideline for Good Clinical Practice and the principles of the Declaration of Helsinki. Patient consent has been obtained for genetic testing and all clinical information reported in this article. Patient information has been coded and deidentified.

### 2.2 Gene panel

The following 19 genes were evaluated using a commercial panel: 8 sarcomere genes with robust association with HCM (*MYH7, MYBPC3, TNNT2, TNNI3, TPM1, MYL2, MYL3, ACTC1*); 7 less prevalent regulatory and structural genes with established association with HCM (*TNNC1, PLN, CSRP3, FLNC, JPH2*); and 4 syndromic and HCM phenocopy genes (*PTPN11, TTR, PRKAG2, LAMP2, GLA, DES*). Specimens were sent in the form of dry blood spots to a certified (ISO 9001:2008) and accredited (PALC-SBPC/ML and DIQC/SBAC) laboratory (*DLE*–Diagnosticos Laboratoriais Especializados, Brazil) with a wide portfolio of national and international proficiency tests (PELM, PNCQ, CDC, PEEC, ERNDIM) for gene panel sequencing.

### 2.3 Procedures and library preparations

Samples were obtained as dried blood spots (DBS) using filter paper and stored at room temperature. DNA extraction was performed using the QIAsymphony Investigator Kit^®^ by Qiagen^®^ and the extracted DNA was stored at 4°C–20 °C. Genomic libraries were prepared following Agilent’s instructions (Agilent Sure Select XT HS and XT Low Input Custom 1–499 kb 96 reactions Design ID: 3,223,981). The DNA libraries underwent qualitative validation through automated gel electrophoresis (TapeStation D1000 Screen Tape) and quantitative validation using fluorescence-based measurement (Qubit™ dsDNA HS Assay Kit). Once validated, the DNA libraries were pooled and hybridized with SureSelect XT HS and XT Low Input kits, which contained custom-designed biotin-labeled oligonucleotide probes for targeting specific genes coding exons +/- 10bps of flanking introns. The target-captured library was prepared using the Agilent SureSelect XT HS and XT Low Input preparation automation guide, with slight modifications for DBS samples, following the manufacturer’s protocol. The concentration of the target-captured DNA fragments, adjusted for size, was determined using the TapeStation D1000 Screen Tape method. For sequencing, either Illumina NextSeq 500 or NovaSeq 6000 sequencers were utilized, employing pair-end and two 150-base pair reads, with a minimum average coverage of 20× to ensure high-quality data. Each sequencing run included a fully characterized positive control.

### 2.4 Sequencing methods and variant annotations

Stringent quality control (QC) procedures were implemented throughout the sequencing process to ensure high-end standards. Mapping and variant QC metrics were calculated and utilized to identify and reevaluate any failed samples. To analyze the sequencing data and extract relevant information, a bioinformatics analysis pipeline was employed. The pipeline involved aligning the sequencing data to the human genome reference sequence version GRCh37 and extracting pertinent details. An in-house software (‘DLE-Tool’) that uses Burrows-Wheeler for alignment (0.7.15), variant calling (single-nucleotide variants and indels) based on mpileup (1.3.1), and Ensembl Variant Effect Predictor (v105) annotation was employed to assess the pathogenicity of all identified variants. In cases with novel variants, the Franklin variant interpretation (Genoox) was specifically employed. A robust methodology was adopted for the detection of single nucleotide variants and small insertions and deletions (indels). Variant information, frequencies and types were analyzed. Allele frequencies were collected from all populations as well as the East Asian subpopulation from a large population database (The Genome Aggregation Database—gnomAD, http://gnomad.broadinstitute.org [version 2.1.1]. The classification of variants followed the guidelines set by the American College of Medical Genetics and Genomics (ACMG) (PMID:25741868). Variants were categorized as pathogenic, likely pathogenic (with a probability greater than 90% of being pathogenic), of uncertain significance, likely benign (with a probability greater than 90% of being benign), or benign.

### 2.5 Variant reporting

The nomenclature follows the updated recommendations of the Human Genome Variation Society (HGVS). Only pathogenic, likely pathogenic and variants of unknown significance were reported. Benign and likely benign variants were not reported. Sanger sequencing confirmed variants of clinical interest that did not fulfill quality requirements.

### 2.6 Statistical analysis

The statistical software package SPSS 22.0 (SPSS Inc.,Chicago, IL) was used for data analyses. Normally and non-normally distributed data were reported as means (standard deviation) and median (interquartile range) respectively. Categorical variables were presented in the form of numbers and percentages. Comparisons between groups were made by Mann-Whitney U-test for continuous variables and χ^2^-test or Fisher’s exact test for categorical variables.

### 2.7 Clinical outcomes and ascertainment

We examined the patients on the occurrence of left ventricular dysfunction and malignant arrhythmias. Left ventricular dysfunction is defined as left ventricular ejection fraction of 50% or less. Measurements of systolic function were validated by cross-referencing between echocardiographic and MRI studies and the images were reviewed by 2 or more experienced cardiologists and/or radiologists. Malignant arrhythmias were defined as aborted sudden cardiac arrest or sustained ventricular arrhythmia or appropriate defibrillator firing. The occurrence of arrhythmic events was confirmed by the presence of electrocardiographic evidence either from standard 12-lead electrocardiogram or from analysis of automated external or implantable defibrillator records. Composite outcome refers to the combined outcomes of left ventricular dysfunction and malignant arrhythmias.

## 3 Results

### 3.1 Clinical characteristics of this HCM cohort

We recruited and analyzed 53 unrelated adult Chinese patients with a clinical diagnosis of HCM in this study. The average age at diagnosis in this cohort is 56 years (SD 15; ranging from 10-85), 35 of the participants were male (66%).

### 3.2 Diagnostic yield of the gene panel in the HCM cohort

**FIGURE 1:**
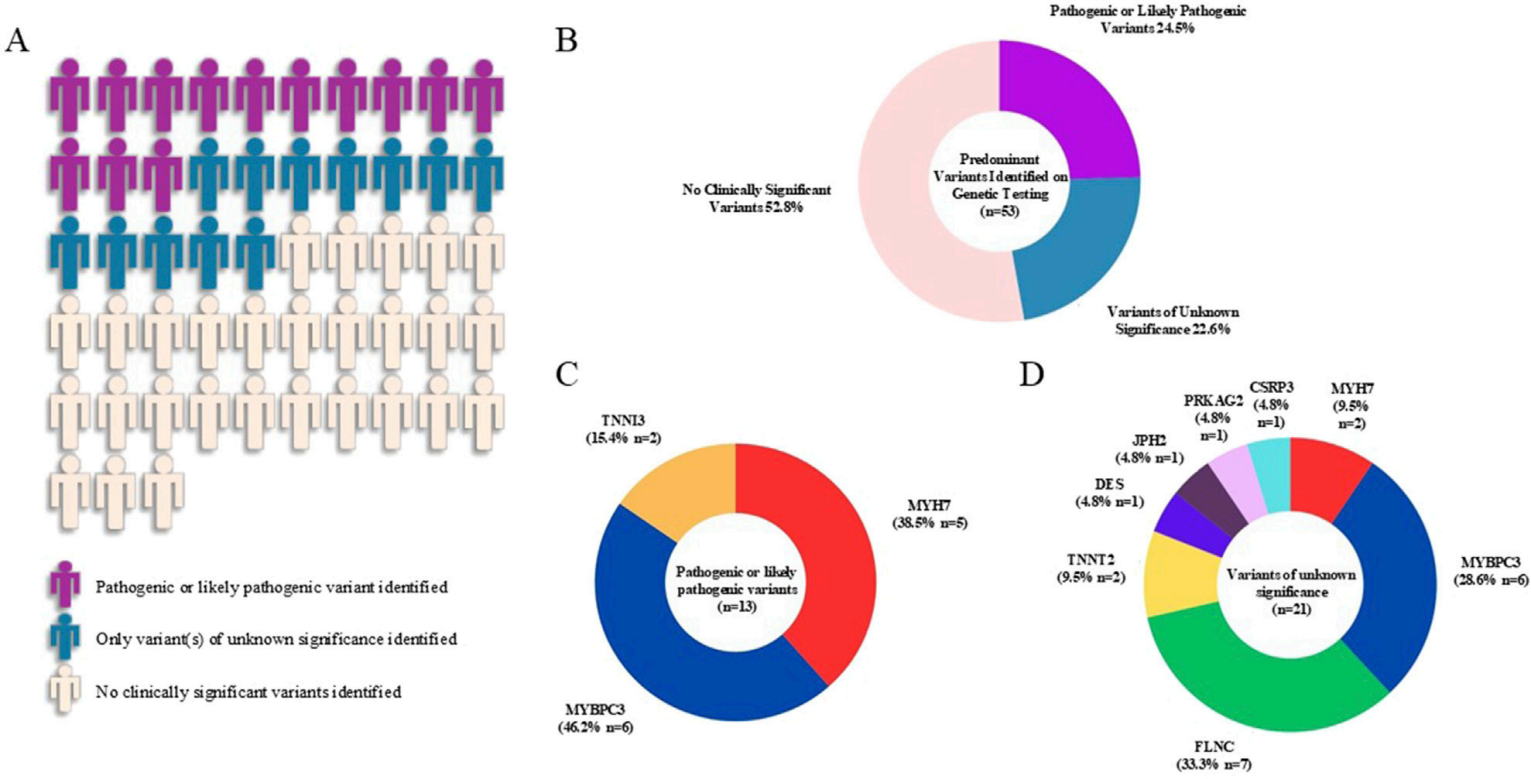
Graphical representation of the yield of genetic testing and frequencies of variants in the hypertrophic cardiomyopathy cohort. Visual representation of the yield of genetic testing was shown in **(A)** and percentages of each variant type were displayed in the pie chart **(B)**. Pathogenic or likely pathogenic variants were identified in three sarcomeric genes (*MYBPC3*, *MYH7*, *TNNI3*) **(C)**. Variants of unknown significance were identified in three core sarcomeric genes (*MYBPC3*, *MYH7*, *TNNT2*), three less prevalent regulatory and structural genes with established association with HCM (*FLNC*, *CSRP3*, *JPH2*) and two syndromic and HCM phenocopy genes (*PRKAG2*, *DES*) **(D)**.

We conducted gene panel testing on the 53 unrelated adult Chinese patients with a clinical diagnosis of HCM. For graphical representation, patients with concomitant P/LP variants and VUS were depicted as having predominantly P/LP variants (purple). Patients with ≥1 VUS and no P/LP were depicted as having predominantly VUS (blue). Overall, P/LP variants were identified in 13 probands (24.5%) and only VUS were identified in 12 probands (22.6%) (Figure 1).

### 3.3 Types of variants identified in the HCM cohort

A total of 13 P/LP variants from 13 patients were found in our HCM cohort (Figures 1A,B). Of these, 6 P/LP variants (46.2%) were identified in *MYBPC3*, 5 (38.5%) in *MYH7* and 2 (15.4%) in *TNNI3* (Figure 1C). Additionally, a total of 21 VUS from 20 patients were found in the cohort (Figure 1D). Among these, 7 VUS (33.3%) were identified in *FLNC*, 6 (28.6%) in *MYBPC3*, 2 (9.5% each) in *MYH7* and *TNNT2* respectively, 1 (4.8% each) in *CSRP3*, *PRKAG2*, *JPH2* and *DES* respectively. Detailed information of all identified variants in this HCM cohort were summarized in Table 2.

**TABLE 2:** Variant information of 25 Chinese unrelated HCM patients with positive genetic testing results.

Study ID	Age at enrollment	Age at clinical diagnosis	Gene	Variant classification	Reference sequence	Genetic variant	Type of mutation	Consequence	Genomic position (GRCh37)	Exon	Allele frequency (all populations)	Allele frequency (east Asian)
Pathogenic or Likely Pathogenic Variants												
1	68	49	*MYH7*	P/LP	NM_000257.4	c.746G>A	Missense	p.Arg249Gln	Chr14:23900677	9 of 40	0	0
11	75	71	*MYH7*	P/LP	NM_000257.4	c.1987C>T	Missense	p.Arg663Cys	Chr14:23896043	18 of 40	0	0
17	78	75	*MYH7*	P/LP	NM_000257.4	c.1987C>T	Missense	p.Arg663Cys	Chr14:23896043	18 of 40	0	0
16	83	65	*MYH7*	P/LP	NM_000257.4	c.2081G>A	Missense	p.Arg694His	Chr14:23895254	19 of 40	0.00001061	0
20	34	10	*MYH7*	P/LP	NM_000257.2	c.2155C>T	Missense	p.Arg719Trp	Chr14:23895180	19 of 40	0.00003185	0
3	48	23	*MYBPC3*	P/LP	NM_000256.3	c.1038_1042 dup	Duplication/Frameshift	p.Met348Thrfs*4	Chr11:47367806	12 of 35	4.017E-06	0.00005565
10	53	50	*MYBPC3*	P/LP	NM_000256.3	c.1038_1042 dup	Duplication/Frameshift	p.Met348Thrfs*4	Chr11:47367806	12 of 35	4.017E-06	0.00005565
15	76	51	*MYBPC3*	P/LP	NM_000256.3	c.1038_1042 dup	Duplication/Frameshift	p.Met348Thrfs*4	Chr11:47367806	12 of 35	4.017E-06	0.00005565
18	42	32	*MYBPC3*	P/LP	NM_000256.3	c.1223 + 1 G>A	Splice donor	P. ?	Chr11:47365042	(Intron)	0.00001478	0.0001351
13	70	49	*MYBPC3*	P/LP	NM_000256.3	c.2864_2865 delCT	Deletion/Frameshift	p.Pro995Argfs Ter95	Chr11:47356633-Chr11:47356634	27 of 35	0	0
19	60	38	*MYBPC3*	P/LP	NM_000256.3	c.3562del	Deletion/Frameshift	p.Val1188*	Chr11:47354182	32 of 35	0	0
7	59	48	*TNNI3*	P/LP	NM_000363.5	c.433C>T	Missense	p.Arg145Trp	Chr19:55665514	7 of 8	0.00001071	0
25	69	54	*TNNI3*	P/LP	NM_000363.5	c.484C>T	Missense	p.Arg162Trp	Chr19:55665463	7 of 8	0.00004016	0.0001669
Variants of Unknown Significance												
9	62	61	*MYH7*	VUS	NM_000257.4	c.911_912delCCinsAG	Indel	p.Thr304Lys	Chr14:23899856-23899857	11 of 40	0	0
14	42	35	*MYH7*	VUS	NM_000257.4	c.1499A>C	Missense	p.Glu500Ala	Chr14:23897788	15 of 40	0	0
19	60	38	*MYBPC3*	VUS	NM_000256.3	c.309G>T	Missense	p.Met103Ile	Chr11:47372150	3 of 35	0	0
13	70	49	*MYBPC3*	VUS	NM_000256.3	c.787G>A	Missense	p.Gly263Arg	Chr11:47369442	7 of 35	0.00008377	0.0005426
6	57	36	*MYBPC3*	VUS	NM_000256.3	c.1000G>A	Missense	p.Glu334Lys	Chr11:47367848	12 of 35	0.0002368	0.003338
21	87	85	*MYBPC3*	VUS	NM_000256.3	c.1000G>A	Missense	p.Glu334Lys	Chr11:47367848	12 of 35	0.0002368	0.003338
23	79	77	*MYBPC3*	VUS	NM_000256.3	c.1000G>A	Missense	p.Glu334Lys	Chr11:47367848	12 of 35	0.0002368	0.003338
15	76	51	*MYBPC3*	VUS	NM_000256.3	c.3005G>A	Missense	p.Arg1002Gln	Chr11:47355293	29 of 35	0.0000599	0
19	60	38	*FLNC*	VUS	NM_001458.5	c.4817T>C	Missense	p.Met1606Thr	Chr7:128488926	28 of 48	0	0
4	77	50	*FLNC*	VUS	NM_001458.5	c.6280G>A	Missense	p.Gly2094Arg	Chr7:128493594	38 of 48	0.00006054	0.0007167
11	75	71	*FLNC*	VUS	NM_001458.5	c.6280G>A	Missense	p.Gly2094Arg	Chr7:128493594	38 of 48	0.00006054	0.0007167
24	72	71	*FLNC*	VUS	NM_001458.5	c.6361 + 3 A>G	Splice region	P. ?	Chr7:128493678	(Intron)	4.008E-06	0.00005563
21	87	85	*FLNC*	VUS	NM_001458.5	c.6683G>A	Missense	p.Arg2228Gln	Chr7:128494226	40 of 48	0.00001234	0.0001126
12	53	51	*FLNC*	VUS	NM_001458.5	c.6689G>A	Missense	p.Arg2230His	Chr7:128494232	40 of 48	0.00009835	0.00005173
22	74	71	*FLNC*	VUS	NM_001458.5	c.7252-14 C>T	Intronic	P. ?	Chr7:128496558	(Intron)	0.00004347	0.0003074
2	49	46	*TNNT2*	VUS	NM_000364.4	c.878G>A	Missense	p.Arg293His	Chr1:201328348	16 of 16	0.0000618	0.000779
12	53	51	*TNNT2*	VUS	NM_000364.4	c.878G>A	Missense	p.Arg293His	Chr1:201328348	16 of 16	0.0000618	0.000779
5	59	57	*PRKAG2*	VUS	NM_016203.4	c.*5C>T	5′Untranslated Region	P. ?	Chr7:151254282	16 of 16	0.00003554	0.0002506
8	56	34	*JPH2*	VUS	NM_020433.5	c.1772_1777dup	Duplication (inframe insertion)	p.Gly591_Ser592dup	Chr20:42744538-42744547	4 of 6	0.00000458	0.00005984
9	62	61	*DES*	VUS	NM_001927.4	c.43C>A	Missense	p.Arg15Ser	Chr2:220283227	1 of 9	4.416E-06	0.00006079
11	75	71	*CSRP3*	VUS	NM_003476.5	c.437G>A	Missense	p.Arg146His	Chr11:19206570	5 of 6	0.00001989	0.00005437

HCM, hypertrophic cardiomyopathy; LP, likely pathogenic; P, pathogenic; VUS, variant of unknown significance.

### 3.4 Allele frequencies in all populations and East Asian subpopulation

**FIGURE 2:**
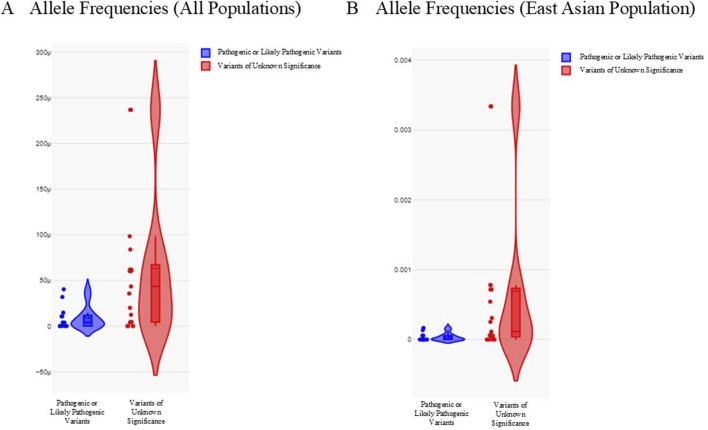
Allele frequencies of pathogenic or likely pathogenic variants and variants of unknown significance in all populations and East Asian subpopulation. **(A)** Violin plot illustrating allele frequencies in all populations. **(B)** Violin plot illustrating allele frequencies in East Asian subpopulation. Pathogenic or likely pathogenic variants were depicted as blue. Variants of unknown significance were depicted as red. Y-axis refers to the allele frequencies.

We analyzed the allele frequencies of all P/LP variants and VUS in our HCM cohort (Figure 2; Table 2). Data were obtained from gnomAD (version 2.1.1). Allele frequencies in all populations and East Asian subpopulation were presented in Figure 2. Compared with VUS, P/LP variants are rarer with allele frequencies confined to <0.00005 in all populations and <0.0002 in East Asian subpopulation. There were overlaps of allele frequencies in P/LP variants and VUS in all populations and East Asian subpopulation, suggesting a fraction of the VUS would be possibly upgraded to P/LP while certain P/LP variants might have reduced penetrance or relatively mild expression with late onset diseases. The allele frequencies were more widely dispersed in the VUS cohort (Figure 2).

### 3.5 MYH7 variants in the HCM cohort

**FIGURE 3:**
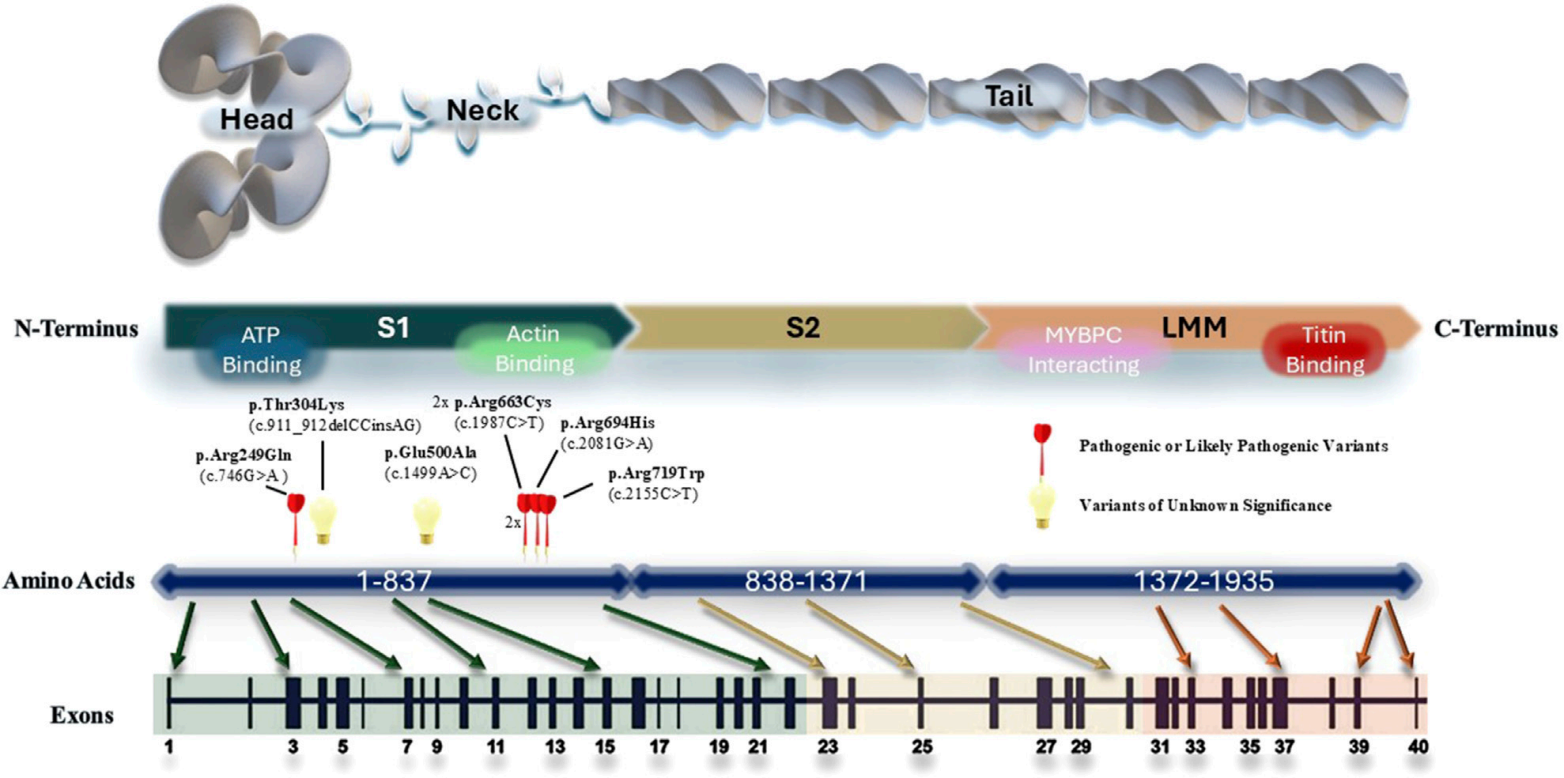
Schematic representation of the *MYH7* gene architecture. All *MYH7* variants identified in the cohort were plotted according to their genomic locations. The regions that are important for protein interactions and ATP binding is marked on the protein structure.

We created a schematic representation of the *MYH7* gene architecture by aligning amino acid and protein domains to the exons (Figure 3). All the *MYH7* variants identified in our HCM cohort clustered around the S1 domain, which corresponds to the head and neck structure of the myosin heavy chain. This was consistent with the known region of cluster of pathogenic *MYH7* variants in HCM identified in Caucasian population (Walsh et al., 2017; Rayment et al., 1995). In addition, we also identified a recurrent pathogenic missense variant (p.R663C) in two unrelated patients. Left ventricular dysfunction occurred in one of the patients.

### 3.6 MYBPC3 variants in the HCM cohort

**FIGURE 4:**
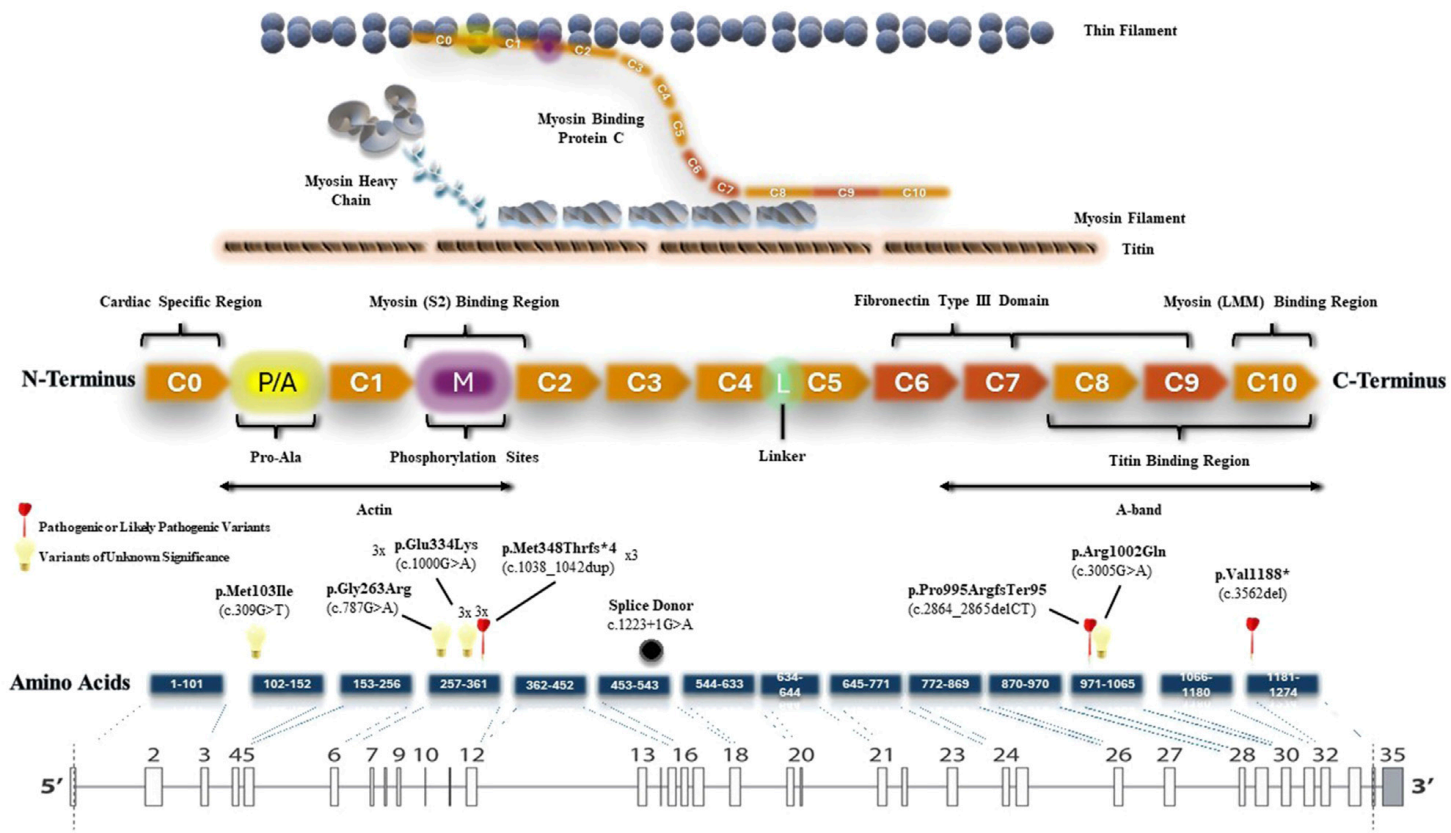
Schematic representation of the *MYBPC3* gene architecture. All *MYBPC3* variants identified in the cohort were plotted according to their genomic locations.

We also created a schematic representation of the *MYBPC3* gene architecture by aligning amino acid and protein domains to the exons (Figure 4). P/LP variants were identified in the M domain (myosin binding region), C8 domain (responsible for titin binding) and C10 domain (myosin binding region). All P/LP *MYBPC3* variants were truncating variants, consistent with its known loss of function mechanism of pathogenicity. In addition to three VUS: c.787G>A (p.Gly263Arg), c.3005G>A (p.Arg1002Gln), c.309G>T (p.Met103Ile), we identified a recurrent pathogenic truncating variant (p.M348Tfs*4) in three unrelated patients and a recurrent missense variant of unknown significance (p.E334K) in another three unrelated patients in our HCM cohort. This recurrent missense VUS has a minor allele frequency of 0.003338 in East Asian subpopulation and 0.0002368 in all populations, corresponding to the VUS with highest MAF in our cohort. Both recurrent variants were located in the myosin (S2) binding region. Among the patients with the recurrent pathogenic truncating variant (p.M348Tfs*4), there were no occurrences of left ventricular dysfunction or malignant arrhythmias. Among the patients with the recurrent missense variant of unknown significance (p.E334K), one of them developed left ventricular dysfunction.

### 3.7 Age at diagnosis stratified by types of variants

**FIGURE 5:**
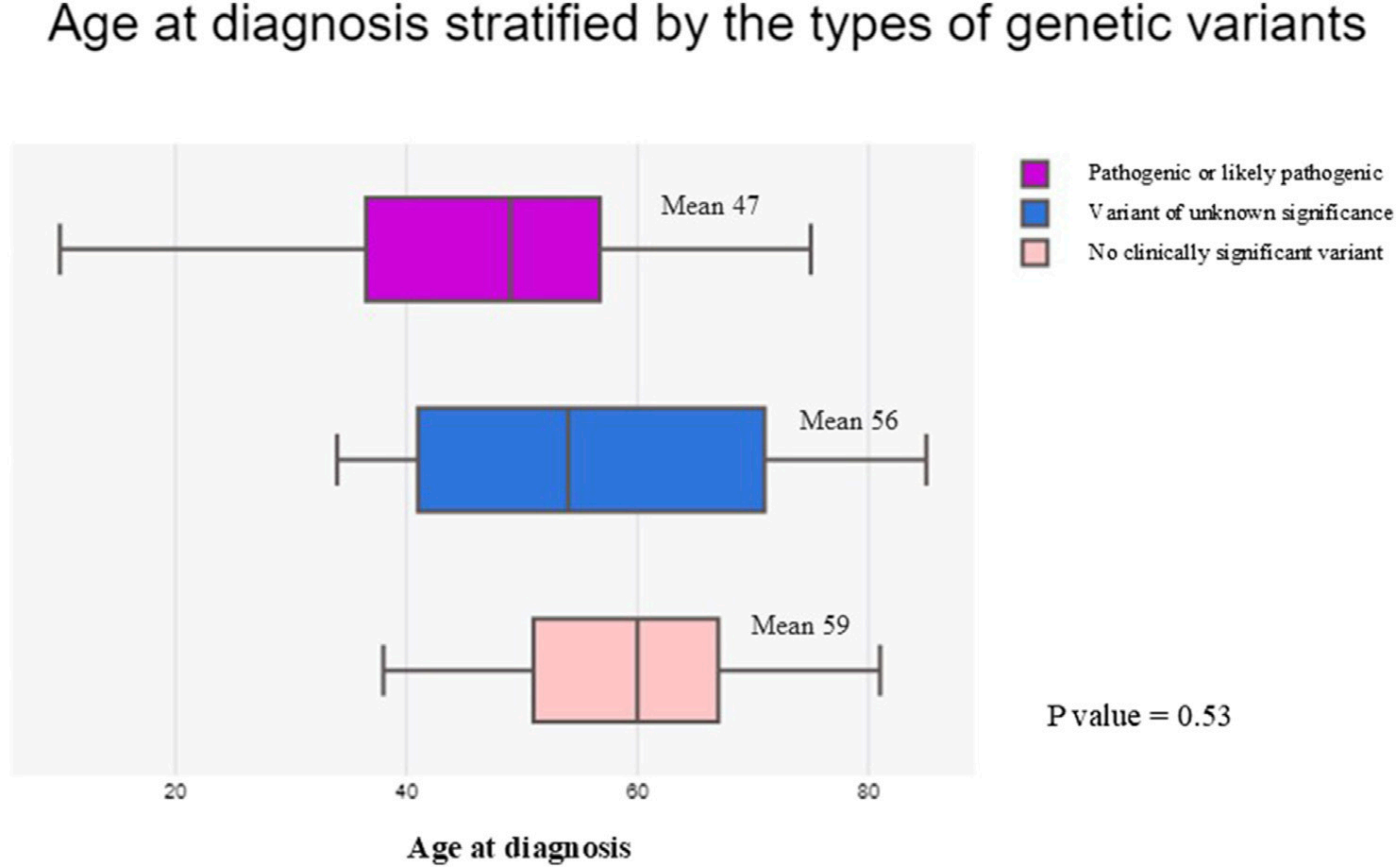
Box plot comparing the age at diagnosis stratified by the types of genetic variants identified in the hypertrophic cardiomyopathy cohort. Patients with pathogenic or likely pathogenic variants were represented by the purple box. Patients with variants of unknown significance were represented by the blue box. Patients with no clinically significant variant identified were represented by the light pink box.

We then examined the relationships between the types of genetic variants identified and the age at diagnosis in our HCM cohort (Figure 5). Patients identified with P/LP variants appeared to receive diagnosis at an earlier age (mean age of 47, IQR = 37-57) compared with patients with VUS (mean age of 56, IQR = 41-71) and patients with no clinically significant variant (mean age of 59, IQR = 51-67). However, the differences in mean age between groups were not statistically significant (p = 0.53), which may be limited by patient number.

### 3.8 Occurrence of major adverse clinical events

We evaluated the association between the occurrence of major adverse clinical events and the types of variants identified in the HCM cohort (Table 3). Patients with P/LP variants were associated with a statistically significant increased risk of developing left ventricular dysfunction compared with patients with VUS or no variants (p = 0.012). There were no statistically significant associations between the types of variants and the occurrence of malignant arrhythmias (p = 0.24) or composite outcome (p = 0.28).

**TABLE 3:** Occurrence of major adverse clinical events stratified by types of variants identified in the hypertrophic cardiomyopathy cohort.

Major adverse clinical events	Pathogenic or likely pathogenic variants n = 13	Variants of unknown significance n = 12	No clinically significant variants n = 28	P Value
Left Ventricular Dysfunction^a^	4	2	0	0.012^a^
Malignant Arrhythmias^b^	0	0	3	0.24
Composite Outcome	4	2	3	0.28

The numbers shown in the table represent the number of patients.

^a^ Left ventricular dysfunction is defined as left ventricular ejection fraction ≤50%.

^b^ Malignant arrhythmias were defined as aborted sudden cardiac arrest or sustained ventricular arrhythmia or appropriate defibrillator therapy.

## 4 Discussion

Our current study provides important insight into the genetic landscape of HCM in Hong Kong Chinese population. The yield of LP/P variants in our Hong Kong Chinese HCM cohort was 24.5%, which was similar but slightly lower than the reported rate of 30%. Similar as previously reported in other population, most LP/P variants were found in *MYBPC3* and *MYH7* (Butters et al., 2020; Alfares et al., 2015; Bonaventura et al., 2021). However, we also found an excess VUS rate of 22.6% in our HCM cohort, compared with 15% as previously described (Alfares et al., 2015). The minor allele frequencies (MAF) of P/LP variants and VUS identified in our HCM cohort were informative and the data were obtained from gnomAD (version 2.1.1). We found that the MAF of the P/LP variants were consistently <0.00005 in all populations and <0.0002 in East Asian subpopulation, which served as reference for variant interpretation. While some of the VUS are rare in the global gnomAD population, their allele frequency in East Asian subpopulation is relatively high for disease-causing variants. The significance of this observation is still unclear, but it may indicate private variants that are specific to the Chinese population.

It is known that disease-causing *MYH7* variants are predominantly missense variants clustering in the head and neck regions, which directly increase the motor function (Walsh et al., 2017). In our cohort, we found similar genomic clustering of *MYH7* variants, which was consistent with the literature (Walsh et al., 2017). In addition, we found two novel *MYH7* VUS located in the head and neck region which have not been previously reported in all population or East Asian subpopulation dataset: c.911_912delCCinsAG (p.Thr304Lys) and c.1499A>C (p.Glu500Ala). The first variant, located in a highly conserved amino acid of *MYH7*, suggests potential pathogenicity as a VUS, although further assessments are necessary. Similarly, the second variant is also predicted to be pathogenic based on *in silico* predictions. The doublet base substitution was found in a 62-year-old woman diagnosed with hypertrophic cardiomyopathy (HCM) at age 61, who reported no significant adverse events. Conversely, the missense variant was identified in a 42-year-old man with HCM diagnosed at 35, who experienced advanced heart failure and left ventricular systolic dysfunction, indicating a more severe clinical outcome.

All pathogenic (P/LP) *MYBPC3* variants identified in our cohort were truncating variants, located throughout the gene, including the M, C8, and C10 domains. These findings support the existing literature that emphasizes loss of function as the primary mechanism of pathogenicity, as well as the distribution of truncating variants in *MYBPC3* (Helms et al., 2020). Furthermore, we have identified six *MYBPC3* VUS in our cohort: c.787G>A (p.Gly263Arg), c.3005G>A (p.Arg1002Gln), c.309G>T (p.Met103Ile) and a recurrent c.1000G>A (p.Glu334Lys). This recurrent *MYBPC3* missense VUS (p.Glu334Lys) has a minor allele frequency of 0.003338 in East Asian subpopulation and 0.0002368 in all populations, corresponding to the VUS with highest MAF in our cohort. The relatively high minor allele frequencies of this variant in East Asian subpopulation likely indicates a benign nature. However, one of the three individuals with this recurrent VUS developed advanced heart failure with left ventricular systolic dysfunction. This may be due to other genetic abnormalities that are not included in this gene panel. The missense VUS c.309G>T (p.Met103Ile) was not found in all population or East Asian subpopulation databases, whereas another VUS c.3005G>A (p.Arg1002Gln) has a minor allele frequency of 0.0000599 in all population but it is absent in East Asian subpopulation. The former VUS was found in patient 19 who also carried a pathogenic frameshift variant c.3562del (p.Val1188*) with no major adverse clinical event at the age of 60. The latter VUS was also identified in patient 15 who carried a concurrent pathogenic frameshift variant c.1038_1042dup (p.Met348Thrfs*4) with no major adverse clinical event at the age of 76.

Moreover, we identified a number of recurrent pathogenic variants in our cohort: a recurrent *MYH7*:c.1987C>T (p.Arg663Cys) pathogenic missense variant, which causes an arginine to cysteine substitution at the amino acid residue 663 and a recurrent *MYBPC3*:c.1038_1042dup (p.Met348Thrfs*4) pathogenic truncating variant, which causes a methionine to threonine substitution at the amino acid residue 348 with a subsequent frameshift. This might represent a co-segregation of alleles or possible founder variant in Hong Kong Chinese ancestry if this finding is replicated in other Asian cohorts. Further evaluation of these recurrent variants in a larger cohort is required. Both individuals with the *MYH7* recurrent pathogenic missense variant were diagnosed after the age of 70; however, one presented with advanced heart failure and left ventricular dysfunction. Among the three individuals with the *MYBPC3* pathogenic truncating variant, diagnoses occurred at ages 23, 50, and 51, respectively, and none experienced any major adverse clinical events.

*TNNI3* mutations account for less than 5% of the solved cases in reported HCM cases. These mutations tend to cluster in exon 7 and 8 (Mogensen et al., 2004; Doolan et al., 2005). In our cohort, 2 out of 13 (15%) P/LP variants were identified in *TNNI3* and both variants (p.R145W and p. R162W) were located in exon 7. p. R145W has been frequently reported in the literature to cause HCM and restrictive cardiomyopathy (RCM). It involves a highly conserved inhibitory region of the cardiac troponin I protein (Lang et al., 2002). One large Korean family described a diverse range of phenotypes from RCM to HCM to near-normal heart observed in family members carrying TNNI3 p. R145W variant (Hwang et al., 2017). No major adverse events observed to date in both of our patients carrying *TNNI3* pathogenic variants. It is possible that incomplete penetrance and age-dependent penetrance might have played a role.

Multiple variants regardless of classification were seen in 7 of 53 individuals (13.2%) in our current HCM cohort, which is more prevalent than that described in the literature (Burns et al., 2017). Previous studies identified multiple rare variants as a risk factor for malignant outcomes in HCM (Burns et al., 2017; Wang et al., 2014). In our cohort, 1 of 7 individuals experienced adverse clinical outcomes, which did not appear to be different from the rest of the cohort, although this result might be limited by the small cohort size in this study. Nonetheless, given the high prevalence of multiple variants in Hong Kong Chinese HCM as highlighted in our study and their potential deleterious effects on clinical outcome, a more extensive dataset on Chinese population could help elucidate further the influence of multiple variants in HCM.

Regarding genotype status and its correlation with major adverse outcomes in HCM, our study found no significant association between the presence of variants and the risk of malignant arrhythmias (p = 0.24) or composite outcomes (p = 0.28). However, we did observe a significant association between the presence of pathogenic or likely pathogenic (P/LP) variants and the risk of left ventricular dysfunction (p = 0.012).

In an analysis from the SHaRe registry (Sarcomeric Human Cardiomyopathy Registry) involving over 4,500 HCM patients, the presence of a sarcomere mutation was shown to predict adverse outcomes, including ventricular arrhythmias, heart failure, and left ventricular dysfunction (Ho et al., 2018). Additionally, a study examining lifetime outcomes of individuals carrying rare variants in sarcomere-encoding genes using UK Biobank data indicated an increased risk of adverse cardiovascular outcomes, primarily related to heart failure, despite a low aggregate penetrance for overt HCM (de Marvao et al., 2021). This suggests a potential link between rare HCM-associated sarcomeric variants and major adverse outcomes, particularly heart failure events.

Conversely, a recent study involving nearly 1,500 patients indicated that genotype status was not a predictor of clinical course, including all-cause mortality, HCM-related mortality, progression to heart failure, or sudden death (Bonaventura et al., 2024). Notably, in all these studies, individuals of Asian ethnicity comprised less than 10% of the study populations. Therefore, it would be valuable to investigate the relationship between genotype status and major adverse cardiac outcomes specifically within the Chinese population.

Regarding clinical implications, this study establishes a foundation for genetic testing in local HCM, showing a considerable yield of nearly 25% through a 19-gene panel, which will be advantageous for subsequent cascade testing among family members. Whilst the impact of variants at the sarcomeric gene level—and even at the level of individual variants—on clinical outcomes and prognosis may be further explored in larger studies, our findings indicate an association between genotype status and adverse clinical outcomes, particularly left ventricular dysfunction, in the Chinese population. Future directions may involve recruiting a larger cohort, gathering family data, revisiting VUS, conducting targeted evaluations of population-specific recurrent variants, and examining genotype-phenotype correlations.

## 5 Limitations

The major limitation of this cohort study is a small sample size, which may limit the generalizability of the findings though it provides novel insights into the unique genetic landscape of HCM within the Hong Kong Chinese population. In addition, as a cross-sectional study, it captures data at a single point which restricts the ability to make causal inferences about relationships between genetic variants and clinical outcomes. Thirdly, family history data were unavailable during this study because clinical genetic services and counseling were not widely accessible through Hong Kong public healthcare. Although family cascade testing was offered in the public sector, access was limited, and clinical screening primarily took place in the private sector, where family clinical data were not easily obtainable from public health records. However, the current work will help increase awareness and potentially test availability in both private and public sectors. We anticipate that a segregation analysis will be included in future studies once family history data become accessible. Moreover, some recognized HCM-associated genes, such as *ALPK3*, *FHOD3*, *FHL1*, and *TRIM63*, were not included in the gene panel during our study. This small gene panel may limit our ability to analyze other genes within the Chinese population and could affect the overall yield of genetic testing.

Future efforts may include a longitudinal follow-up to assess the progression of disease and the long-term clinical outcomes with more comprehensive outcome measurements. Also, the presence of a substantial number of VUS (22.6%) complicates clinical interpretation and management, as the pathogenicity of these variants is uncertain. Further evaluation of these VUS by segregation analysis and haplotype studies should be considered. At the time of our study, we were in the recruitment phase of the Hong Kong Genome Project, the first and largest of its kind in the region, launched by the Hong Kong Genome Institute and the Health Bureau. This population genome data will also enhance our ability to assess the VUS and the founder effect of recurrent variants. Moreover, whilst the recruitment of patients from a single tertiary centre may introduce selection bias, the scale of study centre and the proportion of the population it serves enhance the generalizability of the results.

## 6 Conclusion

Our study provided insight into the genetic landscape of HCM in Hong Kong Chinese population. We identified several recurrent variants and novel variants in our HCM cohort. Patients with P/LP variants were associated with an increased risk of developing left ventricular dysfunction. Future studies on the potential founder effects of these recurrent variants, cumulative effects of multiple variants, and longitudinal follow up of HCM patients would be useful.

## Data Availability

Data supporting this study are not publicly available due to ethical concerns regarding privacy and sensitive information of research participants. Requests for data access should be directed to the corresponding author DL.
