# Correction: Genetic landscape of hypertrophic cardiomyopathy in Hong Kong Chinese population

**DOI:** 10.3389/fgene.2025.1641043

**Published:** 2025-06-25

**Authors:** Derek P. H. Lee, Ye Cao, Lilei Zhang

**Affiliations:** ^1^ Department of Medicine, Queen Elizabeth Hospital, Kowloon, Hong Kong SAR, China; ^2^ Department of Obstetrics and Gynaecology, The Chinese University of Hong Kong, Hong Kong, Hong Kong SAR, China; ^3^ Department of Molecular and Human Genetics, Baylor College of Medicine, Houston, TX, United States

**Keywords:** hypertrophic cardiomyopathy, cardiomyopathy, genetic landscape, population genetics, Hong Kong, Chinese

**Affiliation**: “Department of Obstetrics and Gynaecology, The Chinese University of Hong Kong, Hong Kong, Hong Kong SAR, China” was omitted for author Derek P. H. Lee. This affiliation has now been added for author Derek P. H. Lee.

The original version of this article has been updated.

